# Olmsted syndrome: clinical, molecular and therapeutic aspects

**DOI:** 10.1186/s13023-015-0246-5

**Published:** 2015-03-17

**Authors:** Sabine Duchatelet, Alain Hovnanian

**Affiliations:** INSERM UMR 1163, Laboratory of Genetic skin diseases, Imagine Institute, 2nd floor, 24 bld du Montparnasse, 75015 Paris, France; University Paris Descartes, Sorbonne Paris Cité, Paris, France; Department of Genetics, Necker Enfants Malades Hospital, Paris, France

**Keywords:** Olmsted syndrome, Palmoplantar keratoderma, Periorificial keratotic plaques, Clinical heterogeneity, TRPV3, MBTPS2

## Abstract

Olmsted syndrome (OS) is a rare genodermatosis classically characterized by the combination of bilateral mutilating transgredient palmoplantar keratoderma (PPK) and periorificial keratotic plaques, but which shows considerable clinical heterogeneity. The disease starts usually at birth or in early childhood. About 73 cases have been reported worldwide. OS is observed in both sexes, although male cases are more frequent. The most suggestive symptoms associate PPK with pseudoainhum and periorificial keratotic plaques. Frequently associated features include hair and nail abnormalities, leukokeratosis, corneal default and recurrent infections. Pain and itching are variable but can be severe. Most of reported OS cases are sporadic, although familial cases with different mode of inheritance were also described. Mutations in *TRPV3* (Transient receptor potential vanilloid-3) gene have recently been identified as a cause of autosomal dominant (gain-of-function mutations) or recessive OS. Mutations in *MBTPS2* (membrane-bound transcription factor protease, site 2) gene were identified in a recessive X-linked form. The diagnosis relies mainly on clinical features associating severe PPK and periorificial keratotic plaques, but can be challenging in patients with incomplete phenotype or atypical features. OS has to be differentiated from other severe forms of PPK including Vohwinkel, Clouston, Papillon-Lefèvre or Haim-Munk syndromes, Mal de Meleda, pachyonychia congenita, Tyrosinemia type II and acrodermatitis enteropathica. When differential diagnoses are difficult to exclude, genetic studies are essential to search for a *TRPV3* or *MBTPS2* mutation. However, additional genes remain to be identified. No specific and satisfactory therapy is currently available for OS. Current treatments of hyperkeratosis (mainly emollients, keratolytics, retinoids or corticosteroids), either topical or systemic, are symptomatic and offer only temporary partial relief. Specific management of pain and itching is important to reduce the morbidity of the disease. The disease is debilitating and progressive keratoderma and auto-amputation of digits can prevent patients from grasping and walking, and confine them to a wheelchair. New therapeutic options are therefore crucial and are expected from a better understanding of the disease mechanisms. The use of TRPV3 antagonists would represent such a targeted and potentially powerful strategy.

## Résumé

Le syndrome d’Olmsted (SO) est une génodermatose rare et sévère, classiquement caractérisée par une kératodermie palmo-plantaire (KPP) bilatérale transgrédiente et mutilante associée à des plaques kératosiques périorificielles. Il existe une grande hétérogénéité clinique. Des anomalies des cheveux et des ongles, une leukokératose, des anomalies de la cornée et de fréquentes infections peuvent être associées. La douleur et le prurit sont variables, mais peuvent être sévères. La maladie débute généralement à la naissance ou pendant la petite enfance. Approximativement 73 cas ont été rapportés à travers le monde. La maladie touche les deux genres. La plupart des cas sont sporadiques, bien que des cas familiaux avec différents modes de transmission aient également été décrits. Des mutations du gène *TRPV3* (Transient receptor potential vanilloid-3) ont récemment été identifiées dans les formes autosomiques dominante (mutations gain-de-fonction) et récessive. Des mutations du gène *MBTPS2* (membrane-bound transcription factor protease, site 2) ont été rapportées dans une forme récessive liée à l’X. Le diagnostic est essentiellement clinique, reposant sur l’association d’une KPP sévère et de kératoses périorificielles ; mais il peut être difficile chez les patients ayant une présentation incomplète ou des symptômes atypiques. Le SO doit être différencié des autres KPP sévères telles les syndromes de Vohwinkel, de Clouston, de Papillon-Lefèvre ou de Haim-Munk, le Mal de Meleda, la pachyonychie congénitale, la tyrosinémie de type II et l’acrodermatite entéropathique. Lorsque le diagnostic est difficile à établir, les études génétiques sont essentielles afin de rechercher une mutation des gènes *TRPV3* ou *MBTPS2*. Cependant, d’autres gènes restent à identifier. Il n’existe à ce jour pas de traitement spécifique du SO. Les traitements actuels de l’hyperkératose comprennent essentiellement des émollients, kératolytiques, rétinoïdes et corticoïdes, locaux ou systémiques. La prise en charge spécifique de la douleur et du prurit représente un aspect important du traitement. La KPP massive et l’auto-amputation des doigts peuvent être très invalidants. Le développement de nouveaux traitements est crucial et attendu d’une meilleure compréhension des mécanismes pathologiques de la maladie. L’utilisation d’antagonistes de TRPV3 représenterait une nouvelle option thérapeutique prometteuse.

## Introduction

Olmsted syndrome (OS) is a rare genodermatosis belonging to the heterogeneous group of palmoplantar keratoderma (PPK). OS shows marked clinical and genetic heterogeneity. The aim of this article is to give an updated review of clinical features, genetics, pathological mechanisms, diagnostic criteria, differential diagnosis and current treatment.

## Methods

### Literature review

This review is based on a literature search using PubMed. To identify all publications related to Olmsted syndrome and summarize reported cases, a comprehensive search of PubMed was performed for all studies published prior to December 1, 2014, using the search terms “Olmsted syndrome”, “mutilating palmoplantar keratoderma” or “periorificial keratoderma”. We then checked the bibliography of each article to identify additional references. Altogether, 59 reports containing a total of 73 OS were identified from 1927 until December 1, 2014.

## Review

### Disease name and synonyms

Mutilating palmoplantar keratoderma with periorificial keratotic plaques (ORPHA659, MIM #614594 and #300918)

Olmsted Syndrome (OS)

Palmoplantar and periorificial keratoderma

### Definition

Olmsted syndrome is a rare keratinizing disorder characterized by the combination of bilateral mutilating transgredient palmoplantar keratoderma and periorificial keratotic plaques. This syndrome was first described in 1927 by Olmsted [1]. Since that date, similar cases have been reported in the literature under the heading of Olmsted syndrome.

### Epidemiology

Olmsted syndrome is a rare genodermatosis. The prevalence of the syndrome is unknown. To date, about 73 cases have been reported in the literature (Table 1). Although the number of cases is probably underestimated, the prevalence is likely to be less then 1/1000000.

**Table 1:** **Reported Olmsted syndrome cases in the literature**

**Reference**	**Number of cases**	**Sex**	**Mutation**
Olmsted, 1927 [1]	1	Male	Not reported
Costa, 1962 [2]	1	?	Not reported
Keir, 1967 [3]	1	Female	Not reported
Ruiz-Maldonado et al., 1972 [4]	1	Female	Not reported
Michalowski, 1983 [5]	1	Male	Not reported
Poulin *et al.*, 1984 [6]	1	Male	Not reported
Barnett et al., 1985 [7]	1	Male	Not reported
Harms *et al.*, 1985 [8]	1	Male	Not reported
Rivers *et al.*, 1985 [9]	4 (child, father, paternal aunt and grandmother)	2 females, 2 males	Not reported
Battini *et al.*, 1989 [10]	1	Male	Not reported
Georgii *et al.*, 1989 [11]	1	Female	Not reported
Atherton *et al.*, 1990 [12] and Armstrong et al., 1997 [13]	1 (son of case reported in Keir, 1967 [3])	Male	Not reported
Judge *et al.*, 1991 [14]	1	Male	Not reported
Hausser *et al.*, 1993 [15]	1	?	Not reported
Ueda *et al.*, 1993 [16] and Yoshizaki et al., 2001 [17]	1	Male	Not reported
Lucker et al., 1994 [18]	1	Male	Not reported
Cambiaghi *et al.*, 1995 [19]	2 (twins)	Male	Not reported
Kress *et al.*, 1996 [20] and Raskin and Tu, 1997 [21]	1	Female	Not reported
Dogra *et al.*, 1997 [22]	1	Male	Not reported
Frias-Iniesta *et al.*, 1997 [23]	1	Male	Not reported
Santos *et al.*, 1997 [24]	1	Male	Not reported
Sirka *et al.*, 1999 [25]	1	Male	Not reported
Larregue *et al.*, 2000 [26]	2	Male	Not reported
Fonseca *et al.*, 2001 [27]	1	Female	Not reported
Koch *et al.*, 2001 [28]	1	Female	Not reported
Requena *et al.*, 2001 [29]	1	Male	Not reported
Bergonse *et al.*, 2003 [30]	2	Male	Not reported
Dessureault *et al.*, 2003 [31]	1	Female	Not reported
Ogawa *et al.*, 2003 [32]	1	Female	LOR excluded
Batra and Shah, 2004 [33]	1	Male	Not reported
Inamadar *et al.*, 2004 [34]	1	Male	Not reported
Al-Mutairi *et al.*, 2005 [35]	1	Female	Not reported
Mevorah *et al.*, 2005 [36] and Eytan *et al.*, 2014 [37]	1	Male	TRPV3 p.Trp521Ser
Ziaaddini et al., 2006 [38]	1	Male	Not reported
Ali *et al.*, 2007 [39]	3 (2 brothers)	2 Males, 1 ?	Not reported
Yaghoobi *et al.*, 2007 [40] and Haghighi *et al.*, 2012 [41]	2 (uncle, nephew)	Male	MBTPS2 p.Phe464Ser
Bedard *et al.*, 2008 [42]	2	Female	Not reported
Kumar *et al.*, 2008 [43]	1	Female	Not reported
Tao *et al.*, 2008 [44]	1	Male	Not reported
Nofal *et al.*, 2010 [45]	2 (sister)	Female	Not reported
Vosynioti *et al.*, 2010 [46]	1	Male	Not reported
Tharini *et al.*, 2011 [47]	2	Female	Not reported
Elise Tonoli *et al.*, 2012 [48]	1	Female	Not reported
Lai-Cheong *et al.*, 2012 [49]	1	Male	TRPV3 p.Gly573Ser
Lin *et al.*, 2012 [50]	6	5 females, 1 male	TRPV3 p.Gly573Ser (4 cases), p.Gly573Cys (1 patient) and p.Trp692Gly (1 case)
Tang *et al.*, 2012 [51]	1	Male	Not reported
Attia et al., 2013 [52]	1	Male	Not reported
Danso-Abeam *et al.*, 2013 [53]	1	Female	TRPV3 p.Gly573Ala
Alotaibi *et al.*, 2014 [54]	1	Male	Not reported
Duchatelet *et al.*, 2014a [55]	1	Female	TRPV3 p.Leu673Phe
Duchatelet *et al.*, 2014b [56]	2 (brother)	Male	TRPV3 p.Gly568Cys and p.Gln216_Gly262del
He *et al.*, 2014 [57]	2 (father and son)	Male	TRPV3 p.Gln580Pro
Kariminejad *et al.*, 2014 [58]	1	Male	TRPV3 p.Trp692Cys
Wang *et al.*, 2014 [59]	1	Male	MBTPS2 c.671-9 T > G
**Total**	73	26 Females, 44 Males	14 TRPV3 mutations
			2 MBTPS2 mutations

The disease seems to occur worldwide and is observed in both sexes, although male cases (~63% of OS patients) have been predominantly reported (Table 1).

### Clinical description

The classical clinical phenotype of OS consists of bilateral mutilating palmoplantar keratoderma and periorificial keratotic plaques. However, OS shows high clinical variability and a wide range of inconsistent clinical manifestations have been reported [36,44]. Other features associated with this condition have been described subsequently and continue to be reported. Clinical manifestations described in detail below are not specific for OS, they can be rarely observed and can also vary in severity. In addition, some features observed in OS patients may be fortuitous and not a part of this syndrome. The disease starts usually at birth, in neonatal period or in early childhood, when the child starts to walk and grasp, and worsens over time. The disease has a slow but progressive course.

### Skin

**Figure 1:**
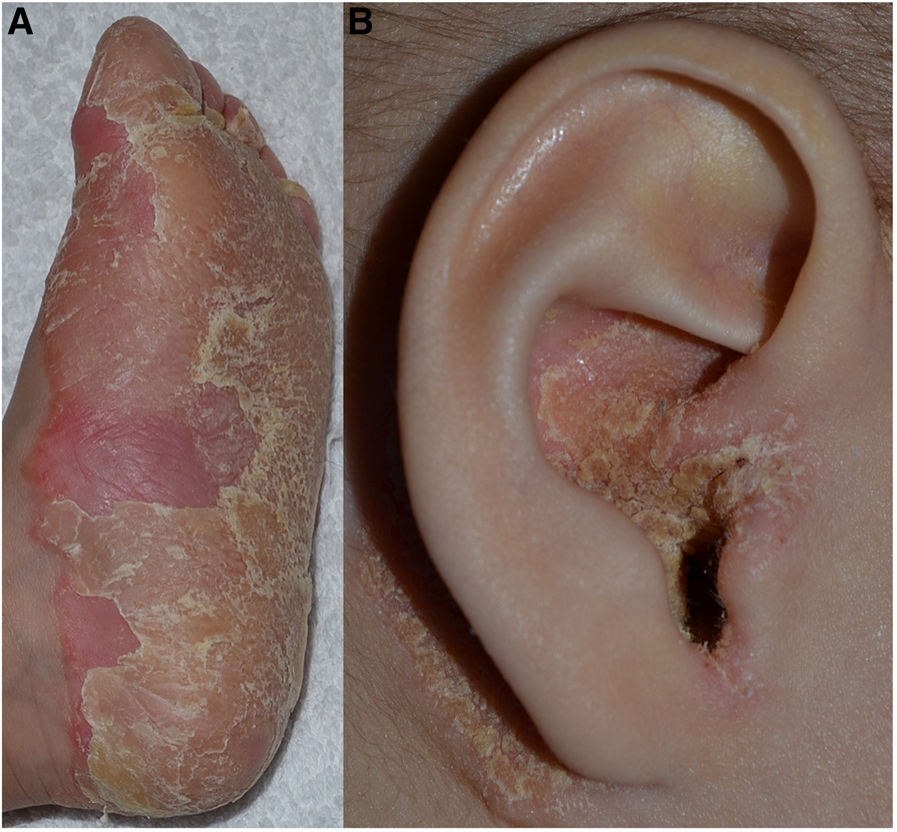
**Clinical features. A.** Diffuse and transgredient, inflammatory plantar keratosis in a 2 year of age child. **B.** Periauricular keratosis in a 2 year of age child.

Initially, the hallmarks for the diagnosis of OS are bilateral mutilating palmoplantar keratoderma (Figure 1A) associated with periorificial keratotic plaques (mouth, nose, eyes, genital, anal, ears, navel) (Figure 1B) [1]. Indeed, progression of the keratoderma may lead to flexion deformities, constrictions of digital bands and even spontaneous digit amputations. However, this pseudoainhum feature is in fact inconsistently reported in OS patient [45]. In addition, in rare cases, periorificial keratotic plaques are absent [55,56]. Keratoderma is initially focal and distributed on the pressure points, gradually extends to most of the surface and becomes diffuse, massive and thicker with time with painful deep fissures. The keratotic lesions are pruritic and can be painful with pressure. Some cases present with focal or punctuate keratoderma [45,57]. A clear improvement of the keratoderma during febrile diseases may occur [19]. Other patients are reported with nonperiorificial keratotic lesions involving the thighs, arms, elbows, knees and intertriginous folds. Hyperkeratotic linear streaks [45,52], follicular keratosis [40], pachyderma, cheilitis [47], ichthyotic lesions or chronic blepharitis [14,23,40] may be also observed.

### Pain and itching

Slight to severe pain with variable intensity is a frequent feature of OS [6,9,18,36,44,45]. Painful PPK is reported in around 50% of OS cases, it can be disabling causing most of the time the patient to avoid walking and grasping. In the less severe patients, only slight pain not interfering with hand and feet activities (walking, writing, grasping objects) is mentioned [45].

Itch has also been reported, although less frequently [36,44]. Pruritus, which can be severe, is mentioned in around 16% of OS cases.

Thus, pain and itch represent major causes of morbidity resulting in insomnia and interfering with walking and grasping.

Atypical OS, with nonmutilating PPK and no periorificial keratotic plaques, associated with erythromelalgia is rarely observed [55,56]. Pain and itching are reported to be particularly severe in uncommon OS patients with erythromelalgia which lead to acute flares of hyperalgesia, severe itch, erythema, vasodilatation and warmth of the legs and extremities (hands, feet and ears) [55,56]. Erythromelalgia is triggered by heat and pain and is reduced by immersion in cold water. In these patients presenting with dysesthesia (tingling or burning sensations) and allodynia with increased sensitivity to thermal and mechanical stimuli, the pain has clearly features of neuropathic pain. The extend to which similar but less severe features could be present in other cases of OS remains to be evaluated.

### Hair and nails

The vast majority of patients present with hair abnormalities including alopecia (diffuse, universal or patchy), hypotrichosis, sparse, thinning, curly, woolly, coarse, dry or easily broken hair [22,36,44,47,51]. Microscopic Hair examination may show pili torti, trichorrhexis nodosa type defects, reduced pigment, as well as longitudinal ridges and transverse fractures of the hair shaft [19,22,36]. Rare OS cases have normal hair.

Sparse and thin eyebrows and eyelashes but also madarosis are also reported [19,40]. In contrast, eyelash trichomegaly is also observed in OS patients [56].

OS patients often show nails abnormalities including dystrophic, lusterless, ridged, rough nails, hyperkeratosis, onychogryphosis, leukonychia, irregular curvatures, onycholysis, paronychia, subungual hyperkeratosis and even absence of nails [22,32,36,43–45,60].

### Oral cavity

Leukokeratosis of the tongue or buccal mucosa have been noted [2,6,11,16,24,27,33,36].

### Sweating

Sweating abnormalities such as palmoplantar hyperhidrosis or anhidrosis, acral hyperhidrosis are reported in OS patients [29,61].

### Eye

Eyes lesions are frequently described in OS patients, including corneal dystrophy, epithelial dysplasia and opacity [10,14,40]. Inflamed lacrimal glands/ducts or meibomian glands dysfunction are also observed.

### Growth

Delayed physical development leading to short stature is frequently described in OS patient [32,36,59].

### Recurrent infections

OS patients are prone to recurrent bacterial or candidal infections in keratotic areas [11,14,20,26,27,30]. Perionychial infections are also mentioned.

### Immunity

The immunological phenotype was assessed in one OS patient and demonstrated immune dysregulation leading to frequent dermal infections, inflammatory infiltrate in the affected areas, hyper IgE production and elevated follicular T cells and eosinophils in the peripheral blood [53]. Immunodeficiency of an unknown origin has been also reported in another patient [11].

### Predisposition to malignancies

OS patients may have a higher susceptibility to develop malignant tumors in keratotic areas such as verrucous carcinoma (a squamous cell carcinoma) and its variant epithelioma cuniculatum located on the sole of the foot, and malignant melanoma [7,15,17,31,32,62].

### Teeth

Abnormal dentition with absence of premolar teeth [6], mammiliations on the free margins of the incisors [34] or peridontal disease leading to premature teeth loss [54] are mentioned.

### Hearing

Hearing loss for high frequencies and congenital deaf-mutism are noted in few OS patients [6,11,29,48].

### Bone

Deformed lower extremities (bow legs) as well as ankylosis (knee and ankles joints), likely resulting from long-term impeded mobility due to painful hyperkeratosis, are described [51]. Osteoporosis and osteolysis of hands and feet [2,3,6,24,26,32] have been also reported.

### Other abnormalities

Other abnormalities including joint laxity [6,11,16,26], primary sclerosing cholangitis [11], intellectual disability [1,12] and haemangioma [35] are also rarely observed in OS patients.

Thus, OS syndrome may result from multiple defects in embryonic structures with a major ectodermal involvement as found in ectodermal dysplasia, with possible minor mesodermal abnormality as manifested by hyperlaxity of joints in some cases.

### Aetiology

#### Genetics

Most reported OS cases are sporadic, although familial cases with different modes of inheritance (dominant or recessive autosomal or X-linked inheritance) were also reported.

**Figure 2:**
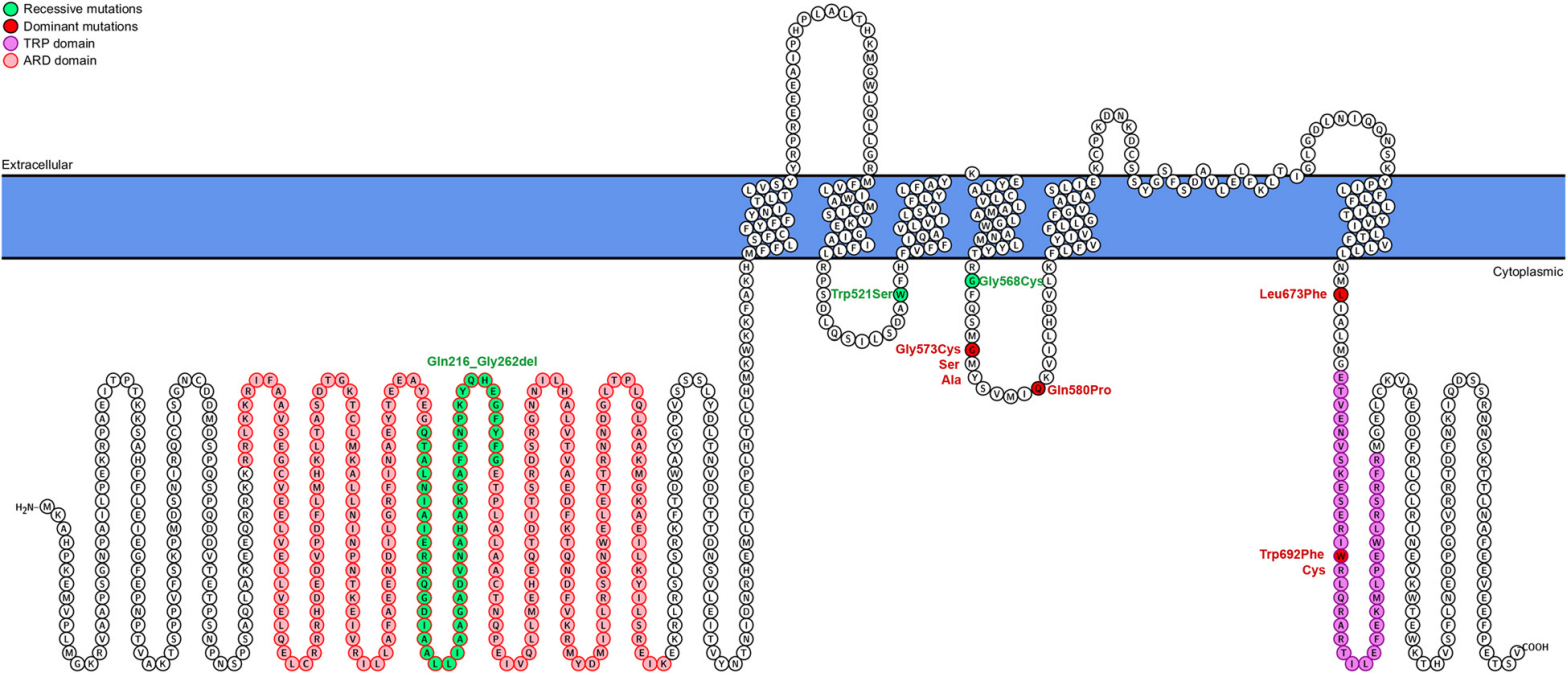
**Schematic structure of TRPV3 and localization of reported OS mutations.** Dominant mutations are indicated in red and recessive mutations in green. Amino acids localized in the ARD (ankyrin repeats domain) and TRP (transient receptor potential) domains are indicated in pink and purple, respectively.

Recently, the genetic basis has been partially elucidated by the identification of mutations in *TRPV3* (Transient receptor potential vanilloid-3) in 14 OS patients with different genetic background (Chinese, Indian, Iranian, Arabic, Caucasian) [37,49,50,53,55–58]. TRPV3 has been reported as a thermosensible cation non selective channel, activated by temperature and several chemical ligands, predominately expressed in keratinocytes, and in sensory neurons [63,64]. TRPV3 is a transmembrane channel belonging to the family of TRP (Transient receptor potential) [63,64] and consists of 6 transmembrane domains with cytoplasmic N and C-termini, assembling as tetramers. *TRPV3* mutations are responsible for autosomal dominant but also recessive OS (Figure 2). To date, 7 dominant mutations (p.Gly573Ser in 5 unrelated patients, p.Gly573Cys, p.Gly573Ala, p.Gln580Pro, p.Leu673Phe, p.Trp692Gly and p.Trp692Cys each in an unique case) have been reported (Figure 2). Several dominant mutations were demonstrated to be gain-of-function mutations leading to an increased intracellular Ca^2+^ [50,57]. Three recessive mutations (p.Trp521Ser in a homozygous state in one patient, and p.Gly568Cys and p.Gln216-Gly262del in a compound heterozygote state in two brothers) were also reported (Figure 2) [37,49,50,53,55–58]. Thus, the Gly573 residue, and Trp692 to a lesser extent, are recurrently mutated. In addition, all mutations, except for two recessive mutations, are located in the S4-5 linker or in the C-terminal part of the protein (Figure 2). The recessive p.Trp521Ser and p.Gln216-Gly262del (resulting from the splicing site mutation c.784 + 1G > A) mutations are located in the S2-3 linker and in the N-terminal part of the protein respectively (Figure 2). OS caused by *TRPV3* mutations shows clinical heterogeneity. Indeed, OS patients with *TRPV3* mutations present either with typical OS hallmarks or incomplete phenotype with atypical features. Genotype-phenotype correlations are difficult to establish at present because of the few reported cases. However, the 5 patients with the same p.Gly573Ser mutation present with a similar phenotype including mutilating PPK with variable severity, hair abnormalities (from dry hair to alopecia) and keratotic plaques (periorificial or only in the natal cleft). In the other hand, other mutations, located in the same part or in different domains of the protein, are associated with atypical features (Figure 2). The p.Leu673Phe (dominant), p.Gly568Cys (recessive) and p.Gln216-Gly526del (recessive) mutations lead to atypical OS, with nonmutilating PPK and no periorificial keratotic plaques, associated with erythromelalgia [55,56]. The patient with the p.Gly573Ala presents with immune dysregulation [53]. The 2 related patients with the p.Gln580Pro mutation have a very mild phenotype with only focal PPK even at 37-year-old for the father [57].

Mutations in *MBTPS2* (membrane-bound transcription factor protease, site 2) gene (p.Phe464Ser and c.671-9 T > G), encoding a zinc metalloprotease essential for cholesterol homeostasis and endoplasmic reticulum stress response, have also been reported in recessive X-linked OS [41,59]. Mutations in *MBTPS2* also lead to IFAP (Ichthyosis follicularis - atrichia - photophobia) syndrome with or without BRESHECK syndrome (MIM #308205, ORPHA2273) [65]. BRESHECK syndrome is a multiple congenital malformation characterized by intellectual disability, brain anomalies, Hirschsprung disease, corneal opacifications, kidney dysplasia, cryptorchidism, cleft palate, and skeletal malformations, particularly of the vertebrae. The OS patient with the c.671-9 T > G mutation also presents with classic IFAP syndrome [59], whereas previously reported patients with the same mutation in *MBTPS2* has no OS features. The mechanism by which *MBTPS2* mutations lead to IFAP or OS is currently unclear.

There is no obvious clinical difference between OS caused by *TRPV3* and *MBTPS2* mutations despite the mode of inheritance. In addition, clinical variability is observed even in patients from the same family or unrelated patients harbouring the same mutation, suggesting the implication of modifier genes, epigenetics and/or environmental factors.

Finally, some patients do not carry detectable mutations in either gene, indicating further genetic heterogeneity.

#### Pathogenesis

The pathogenesis of OS is poorly understood. However, a defect in the expression of mature epidermal keratins (1 and 10) and persistence of basal keratins (5 and 14) was reported [20,21,27,45], as well as increased expression of Ki-67, a mitotic activity marker in basal and suprabasal layers [29,44,48]. Thus, involved areas remain in an immature state and continue to proliferate in an improper fashion. This excessive epithelial proliferation may result in hyperkeratosis. The mechanism by which *TRPV3* mutations lead to OS pathogenesis is unknown. Functional studies of four *TRPV3* mutations (p.Gly573Ser, p.Gly573Cys, p.Trp692Gly and p.Gln580Pro) using patch clamp analysis on transfected HEK293 cells expressing TRPV3 mutants were performed [50,57]. These studies have shown that these mutations lead to much larger inward currents probably because of the constitutive opening of the mutants [50]. Thus, these mutations are gain-of-function mutations and result in elevated intracellular Ca^2+^ inducing increased apoptosis, suggesting that enhanced cell-death could drive OS [50]. In addition, murine models have shown that TRPV3 is also associated with TGF-α/EGFR signaling, which plays a role in keratinocyte differentiation control [66]. Reported immune dysregulation in OS suggests a primary (as TRPV3 is expressed by Langerhans cells) or secondary role of immunological processes in OS pathogenesis [53]. Transcriptomic and proteomic investigation in patients with the p.Gln580Pro mutation, leading only to focal PPK, showed decreased expression of proteins involved in keratinocyte differentiation, in gap junction, tight junction and desmosome formation, but increased expression of proteins involved in keratinocyte proliferation and cell death [57]. Thus, the *TRPV3* p.Gln580Pro gain-of-function mutation disrupts the balance between keratinocyte proliferation and differentiation, and compromises cell adhesion. In addition, previous studies on murine models have also demonstrated that TRPV3 mediates release of pro-inflammatory cytokines including interleukin IL-1α [67], an important mediator of cutaneous inflammation, but also prostaglandin E2 [68]. Of note, TRPV3 is expressed in nociceptive neurons. Thus, it is also likely that TRPV3 over-activation leads to local overproduction of inflammatory cytokines which could affect the somatosensory system, leading to a peripheral neuropathic pain.

### Diagnosis and diagnostic methods

Olmsted syndrome diagnosis relies on the clinical presentation. Initially, the diagnostic hallmarks of OS are the association of bilateral transgredient mutilating palmoplantar keratoderma and periorificial keratotic plaques (mouth, nose, eyes, genital, anal, ears, navel) which allows exclusion of other hyperkeratotic related disorders. However, subsequent reports showed that this full-blown OS is not always observed, making the diagnosis challenging for patients with partial cutaneous expression of OS. Unfortunately, the histopathology of skin sections shows epidermal hyperplasia, orthohyperkeratosis, focal parakeratosis, hypergranulosis, acanthosis, and inflammatory infiltration in upper dermis, which are unspecific and of limited use to orientate the diagnosis. In the absence of specific biologic markers, molecular genetics is the most powerful approach to establish the diagnosis when the clinical presentation is unusual.

### Differential diagnosis

The diagnosis of Olmsted syndrome is supported by clinical criteria that require the exclusion of other diseases. OS has to be differentiated from other severe forms of PPK, such as Vohwinkel syndrome (MIM#124500 and 604117, ORPHA494 and ORPHA79395), Mal de Meleda (MIM#248300, ORPHA87503), Clouston syndrome (MIM#129500, ORPHA189), Papillon-Lefèvre syndrome (MIM#245000, ORPHA678), pachyonychia congenita (MIM#167200 and 167210, ORPHA2309), Tyrosinemia type II (MIM#276600, ORPHA28378) and Haim-Munk syndrome (MIM#245010, ORPHA2342) (Table 2) [52]. The periorificial involvement is the unique characteristic of OS that allows exclusion of the above syndromes. However, because of periorificial involvement, OS is easily confused with acrodermatitis enteropathica (MIM#201100, ORPHA37) which however can be excluded by measurement of zinc levels. Indeed, low plasma level of zinc is diagnostic of acrodermatitis enteropathica which shows improvement after oral zinc therapy, unlike OS. Thus, the full-blown OS with its typical association of mutilating bilateral PPK and periorificial keratotic plaques is distinctive enough to lead to the correct diagnosis.

**Table 2:** **Differential diagnosis and underlying molecular basis**

**Disease**	**Gene**	**Mode of inheritance**
Vohwinkel syndrome	*GJB2, LOR*	Autosomal dominant
Mal de Meleda	*SLURP1*	Autosomal recessive
Papillon-Lefèvre syndrome	*CTSC*	Autosomal recessive
Clouston syndrome	*GJB6*	Autosomal dominant
Pachyonychia congenita	*KRT6A, KRT6B, KRT16 and KRT17*	Autosomal dominant
Tyrosinemia type II	*TAT*	Autosomal recessive
Haim-Munk syndrome	*CTSC*	Autosomal recessive
Acrodermatitis enteropathica	*SLC39A4*	Autosomal recessive

However, the diagnosis becomes challenging for patients with partial and moderate cutaneous expression because of clinical overlap with other PPK mentioned above. In this case, OS diagnosis should be considered and molecular genetics and the mode of inheritance may help to discriminate these disorders (Table 2). The difficult clinical and histopathological distinction between different PPK makes genetic testing important in the diagnostic approach to these patients.

### Genetic counselling and antenatal diagnosis

Most reported OS cases are sporadic, although familial cases were also reported. For familial cases, the mode of inheritance of OS has not been clearly established. Thus, different modes of inheritance have been suggested including dominant or recessive autosomal pattern but also dominant or recessive X-linked inheritance.

As with all severe genetic disorders, genetic counselling and psychological support are appropriate and requested for the families, to discuss the aetiology, the probability of occurrence in future pregnancies, the possibilities of early prenatal diagnosis and current treatment available.

### Management including treatment

There is currently no specific nor satisfactory treatment for OS. Skin lesions are usually refractory to therapy. Different treatments have been tried with varying success to reduce hyperkeratosis. Topical treatments including emollients (white petrolatum), keratolytics (urea, salicylic acid), wet dressing, boric acid, tar, retinoid acid, shale oil, corticosteroids, steroids are attempted with poor to moderate improvement. In several OS patients, systemic retinoids (acitretin, etretinate), corticosteroid or methotrexate were used with poor to moderate relief [9,15,16,51]. Repeated partial excision of palmoplantar keratosis or full-thickness excision with skin grafting have been also performed for few patients but was usually followed by recurrence of the hyperkeratosis after initial improvement [42].

Until now, treatments either topical or systemic only offer temporary partial symptomatic relief of pain and fissures by reducing the thickness keratotic palmoplantar skin lesions. Specific treatments of pain and itching are essential to reduce the morbidity of the disease. Painkillers and topical lidocaine can be effective, but for patients with severe pain and especially when erythromelalgia and/or neuropathic pain are present, anti-inflammatory drugs, anticonvulsants (carbamazepine, gabapentin, pregabalin), tricyclic antidepressants (amitriptyline, nortriptyline) and lastly opioid analgesics (tramadol) are required to alleviate the pain of these patients. Prolonged soaking of the affected parts in cold water may reduce pain. However, these treatments are ineffective in arresting the course of the disease. The identification of TRPV3 gain-of-function mutations indicates that TRPV3 antagonists may be an effective therapeutic approach to treat the OS patients harbouring such mutations.

### Prognosis

The natural history of the disease can lead to severe morbidities. Progressive keratoderma and auto-amputation of digits worsens the disability by preventing patients from grasping and walking, and can confine them to a wheelchair. Severe pruritus and pain increase the discomfort and can result in insomnia. Corneal dystrophy can cause blindness. In addition, OS patients may have a higher susceptibility to develop tumors in keratotic areas. No reduced life expectancy has been reported.

### Unresolved questions

There have been recently many major advances in our understanding of the genetic basis of OS, with the identification of *TRPV3* and *MBTPS2* mutations, although other causal genes remain to be identified. Several questions remain unanswered with regards to the great variability of clinical expression of the disease, the lack of phenotype/genotype correlation and the exact pathophysiological mechanism(s) underlying OS. The broad spectrum of OS clinical features raises the question whether atypical reported cases really belong to OS. Mutations within *TRPV3* can cause both typical and atypical OS cases. Thus, OS may be a heterogeneous syndrome with variable clinical features.

## Conclusion

For 87 years after its first description, around 73 OS cases have been reported, increasing the spectrum of clinical features associated with this condition and disclosing variable clinical expression. OS diagnosis may be challenging in the absence of the full-blown cutaneous phenotype associating bilateral mutilating PPK and periorificial keratotic plaques. The recent advances in the genetics of OS has lead to a better understanding of the aetiology of the disease and make possible the use of diagnostic genetic testing. Currently available drugs are nonspecific and poorly effective. The identification of the molecular basis of OS will permit the development of specific therapeutic strategies such as TRPV3 antagonists, and better patient management. However, other genes remain to be identified and next generation sequencing is likely to permit to further elucidate the genetics of OS and its clinical spectrum in patients with no *TRPV3* or *MBTPS2* mutation.

## Abbreviations

OS: Olmsted syndrome
PKK: Palmoplantar keratoderma
TRPV3: Transient receptor potential vanilloid-3
MBTPS2: Membrane-bound transcription factor protease, site 2
IFAP: Ichthyosis follicularis atrichia photophobia

## References

[1] Olmsted HC (1927). Keratodermia palmaris et plantaris congenitalis: report of a case showing associated lesions of unusual location. Am J Dis Child.

[2] Costa O (1962). Acroceratosis. PhD Thesis.

[3] Keir M (1967). Keratodermia palmaris et plantaris. Br J Dermatol.

[4] Ruiz-Maldonado R, Lozano-Ferral N (1972). Mutilating palmo-plantar hyperkeratosis with alopecia and erythematous inguinal and perianal lesions. Int J Dermatol.

[5] Michalowski R (1983). Erythrokeratoderma periorificialis with involvement of the extremities. Hautarzt.

[6] Poulin Y, Perry HO, Muller SA (1984). Olmsted syndrome–congenital palmoplantar and periorificial keratoderma. J Am Acad Dermatol.

[7] Barnett JH, Estes SA (1985). Multiple epitheliomata cuniculata occurring in a mutilating keratoderma. Cutis.

[8] Harms M, Bergues J, Saurat JH (1985). Syndrome de Olmsted. Dermatologica.

[9] Rivers JK, Duke EE, Justus DW (1985). Etretinate: management of keratoma hereditaria mutilans in four family members. J Am Acad Dermatol.

[10] Battini M, Moretti S, Giovannucci Uzielli M, Happle R. Olmsted syndrome: a case associated with severe corneal opacities. Caputo R, Gelmetti C, editors Milan, Italy:Proceedings of the 5th International Congress of Pediatric Dermatology; July 11–15 1989.

[11] Georgii A, Przybilla B, Schmoeckel C (1989). Olmstedt syndrome–associated with primary sclerosing cholangitis and immune deficiency of uncertain origin. Hautarzt.

[12] Atherton DJ, Sutton C, Jones BM (1990). Mutilating palmoplantar keratoderma with periorificial keratotic plaques (Olmsted’s syndrome). Br J Dermatol.

[13] Armstrong AP, Percival N (1997). Olmsted’s syndrome. J R Soc Med.

[14] Judge MR, Misch K, Wright P, Harper JI (1991). Palmoplantar and perioroficial keratoderma with corneal epithelial dysplasia: a new syndrome. Br J Dermatol.

[15] Hausser I, Frantzmann Y, Anton-Lamprecht I, Estes S, Frosch PJ (1993). Olmsted syndrome. Successful therapy by treatment with etretinate. Hautarzt.

[16] Ueda M, Nakagawa K, Hayashi K, Shimizu R, Ichihashi M (1993). Partial improvement of Olmsted syndrome with etretinate. Pediatr Dermatol.

[17] Yoshizaki Y, Kanki H, Ueda T, Ichihashi M, Ueda M (2001). A further case of plantar squamous cell carcinoma arising in Olmsted syndrome. Br J Dermatol.

[18] Lucker GP, Steijlen PM (1994). The Olmsted syndrome: mutilating palmoplantar and periorificial keratoderma. J Am Acad Dermatol.

[19] Cambiaghi S, Tadini G, Barbareschi M, Caputo R (1995). Olmsted syndrome in twins. Arch Dermatol.

[20] Kress DW, Seraly MP, Falo L, Kim B, Jegasothy BV, Cohen B (1996). Olmsted syndrome. Case report and identification of a keratin abnormality. Arch Dermatol.

[21] Raskin CA, Tu JH (1997). Keratin expression in Olmsted syndrome. Arch Dermatol.

[22] Dogra D, Ravindraprasad JS, Khanna N, Pandhi RK (1997). Olmsted syndrome with hypotrichosis. Indian J Dermatol Venereol Leprol.

[23] Frias-Iniesta J, Sanchez-Pedreno P, Martinez-Escribano JA, Jimenez-Martinez A (1997). Olmsted syndrome: report of a new case. Br J Dermatol.

[24] Santos OL, Amorim JH, Voloch K, Gomes M, Ramos-e-Silva M, Pereira Junior AC (1997). The Olmsted syndrome. Int J Dermatol.

[25] Sirka CS, Ramam M, Mittal R, Khaitan BK, Verma KK (1999). Olmsted syndrome. Indian J Dermatol Venereol Leprol.

[26] Larregue M, Callot V, Kanitakis J, Suau AM, Foret M (2000). Olmsted syndrome: report of two new cases and literature review. J Dermatol.

[27] Fonseca E, Pena C, Del Pozo J, Almagro M, Yebra MT, Cuevas J (2001). Olmsted syndrome. J Cutan Pathol.

[28] Koch P, Reinhold U, Tilgen W (2001). Olmsted syndrome. Ann Dermatol Venereol.

[29] Requena L, Manzarbeitia F, Moreno C, Izquierdo MJ, Pastor MA, Carrasco L (2001). Olmsted syndrome: report of a case with study of the cellular proliferation in keratoderma. Am J Dermatopathol.

[30] Bergonse FN, Rabello SM, Barreto RL, Romiti R, Nico MM, Aoki V (2003). Olmsted syndrome: the clinical spectrum of mutilating palmoplantar keratoderma. Pediatr Dermatol.

[31] Dessureault J, Poulin Y, Bourcier M, Gagne E (2003). Olmsted syndrome-palmoplantar and periorificial keratodermas: association with malignant melanoma. J Cutan Med Surg.

[32] Ogawa F, Udono M, Murota H, Shimizu K, Takahashi H, Ishida-Yamamoto A (2003). Olmsted syndrome with squamous cell carcinoma of extremities and adenocarcinoma of the lung: failure to detect loricrin gene mutation. Eur J Dermatol.

[33] Batra P, Shah N (2004). Olmsted syndrome–a rare syndrome with oral manifestations. Oral Surg Oral Med Oral Pathol Oral Radiol Endod.

[34] Inamadar AC, Palit A, Athanikar SB, Sampagavi VV, Deshmukh NS (2004). What syndrome is this? Olmsted syndrome. Pediatr Dermatol.

[35] Al-Mutairi N, Sharma AK, Nour-Eldin O, Al-Adawy E (2005). Olmsted syndrome: report of a new case with unusual features. Clin Exp Dermatol.

[36] Mevorah B, Goldberg I, Sprecher E, Bergman R, Metzker A, Luria R (2005). Olmsted syndrome: mutilating palmoplantar keratoderma with periorificial keratotic plaques. J Am Acad Dermatol.

[37] Eytan O, Fuchs-Telem D, Mevorach B, Indelman M, Bergman R, Sarig O (2014). Olmsted syndrome caused by a homozygous recessive mutation in TRPV3. J Invest Dermatol.

[38] Ziaaddini H, Shamsadini S (2006). Olmsted syndrome associated with somatic delusion. Iran J Med Sci.

[39] Ali ME, Sikdar AU, Akhtar N, Islam ZM (2007). Olmsted syndrome. Mymensingh Med J.

[40] Yaghoobi R, Omidian M, Sina N, Abtahian SA, Panahi-Bazaz MR (2007). Olmsted syndrome in an Iranian family: report of two new cases. Arch Iran Med.

[41] Haghighi A, Scott CA, Poon DS, Yaghoobi R, Saleh-Gohari N, Plagnol V (2012). A missense mutation in the MBTPS2 gene underlies the X-linked form of Olmsted syndrome. J Invest Dermatol.

[42] Bedard MS, Powell J, Laberge L, Allard-Dansereau C, Bortoluzzi P, Marcoux D (2008). Palmoplantar keratoderma and skin grafting: postsurgical long-term follow-up of two cases with Olmsted syndrome. Pediatr Dermatol.

[43] Kumar P, Sharma PK, Kar HK (2008). Olmsted syndrome. Indian J Dermatol.

[44] Tao J, Huang CZ, Yu NW, Wu Y, Liu YQ, Li Y (2008). Olmsted syndrome: a case report and review of literature. Int J Dermatol.

[45] Nofal A, Assaf M, Nassar A, Nofal E, Shehab M, El-Kabany M (2010). Nonmutilating palmoplantar and periorificial kertoderma: a variant of Olmsted syndrome or a distinct entity?. Int J Dermatol.

[46] Vosynioti V, Kosmadaki M, Tagka A, Katsarou A (2010). A case of Olmsted syndrome. Eur J Dermatol.

[47] Tharini GK, Hema N, Jayakumar S, Parveen B (2011). Olmsted syndrome: report of two cases. Indian J Dermatol.

[48] Elise Tonoli R, De Villa D, Hubner Frainer R, Pizzarro Meneghello L, Ricachnevsky N, de Quadros M (2012). Olmsted syndrome. Case Rep Dermatol Med.

[49] Lai-Cheong JE, Sethuraman G, Ramam M, Stone K, Simpson MA, McGrath JA (2012). Recurrent heterozygous missense mutation, p.Gly573Ser, in the TRPV3 gene in an Indian boy with sporadic Olmsted syndrome. Br J Dermatol.

[50] Lin Z, Chen Q, Lee M, Cao X, Zhang J, Ma D (2012). Exome sequencing reveals mutations in TRPV3 as a cause of Olmsted syndrome. Am J Hum Genet.

[51] Tang L, Zhang L, Ding H, Wang X, Wang H (2012). Olmsted syndrome: a new case complicated with easily broken hair and treated with oral retinoid. J Dermatol.

[52] Attia AM, Bakry OA (2013). Olmsted syndrome. J Dermatol Case Rep.

[53] Danso-Abeam D, Zhang J, Dooley J, Staats KA, Van Eyck L, Van Brussel T (2013). Olmsted syndrome: exploration of the immunological phenotype. Orphanet J Rare Dis.

[54] Alotaibi AK, Alotaibi MK, Alsaeed S, Alyahya A, Shuler CF. Olmsted syndrome with oral involvement, including premature teeth loss. Odontology. 2014. doi:10.1007/s10266-014-0148-3. 10.1007/s10266-014-0148-3 24474548

[55] Duchatelet S, Pruvost S, de Veer S, Fraitag S, Nitschke P, Bole-Feysot C (2014). A new TRPV3 missense mutation in a patient with Olmsted syndrome and Erythromelalgia. JAMA Dermatol.

[56] Duchatelet S, Guibbal L, de Veer S, Fraitag S, Nitschke P, Zarhrate M (2014). Olmsted syndrome with erythromelalgia caused by recessive TRPV3 mutations. Br J Dermatol.

[57] He Y, Zeng K, Zhang X, Chen Q, Wu J, Li H (2015). A gain-of-function mutation in TRPV3 causes focal Palmoplantar Keratoderma in a Chinese family. J Invest Dermatol.

[58] Kariminejad A, Barzegar M, Abdollahimajd F, Pramanik R, McGrath JA (2014). Olmsted syndrome in an Iranian boy with a new de novo mutation in TRPV3. Clin Exp Dermatol.

[59] Wang HJ, Tang ZL, Lin ZM, Dai LL, Chen Q, Yang Y (2014). Recurrent splice-site mutation in MBTPS2 underlying IFAP syndrome with Olmsted syndrome-like features in a Chinese patient. Clin Exp Dermatol.

[60] Ferone G, Mollo MR, Thomason HA, Antonini D, Zhou H, Ambrosio R (2013). p63 control of desmosome gene expression and adhesion is compromised in AEC syndrome. Hum Mol Genet.

[61] Perry HO, Su WP (1995). Olmsted syndrome. Semin Dermatol.

[62] Yamamoto T, Azukizawa H, Tadokoro T (1999). A case of Olmsted syndrome associated with a plantar verrucous carcinoma. Jpn J Dermatol.

[63] Nilius B, Biro T (2013). TRPV3: a ’more than skinny’ channel. Exp Dermatol.

[64] Nilius B, Biro T, Owsianik G (2013). TRPV3: time to decipher a poorly understood family member!. J Physiol.

[65] Megarbane H, Megarbane A (2011). Ichthyosis follicularis, alopecia, and photophobia (IFAP) syndrome. Orphanet J Rare Dis.

[66] Cheng X, Jin J, Hu L, Shen D, Dong XP, Samie MA (2010). TRP channel regulates EGFR signaling in hair morphogenesis and skin barrier formation. Cell.

[67] Xu H, Delling M, Jun JC, Clapham DE (2006). Oregano, thyme and clove-derived flavors and skin sensitizers activate specific TRP channels. Nat Neurosci.

[68] Huang SM, Lee H, Chung MK, Park U, Yu YY, Bradshaw HB (2008). Overexpressed transient receptor potential vanilloid 3 ion channels in skin keratinocytes modulate pain sensitivity via prostaglandin E2. J Neurosci.

